# Supplementary material for: The plastid cysteine synthase complex regulates ABA biosynthesis and stomatal closure in Arabidopsis
Source: Nat Commun. 2025 Oct 8;16:8960. doi: 10.1038/s41467-025-64705-3 (PMC12508444; doi:10.1038/s41467-025-64705-3)
Supplement: Supplementary file 1 — Supplementary Information [file 41467_2025_64705_MOESM1_ESM.pdf]

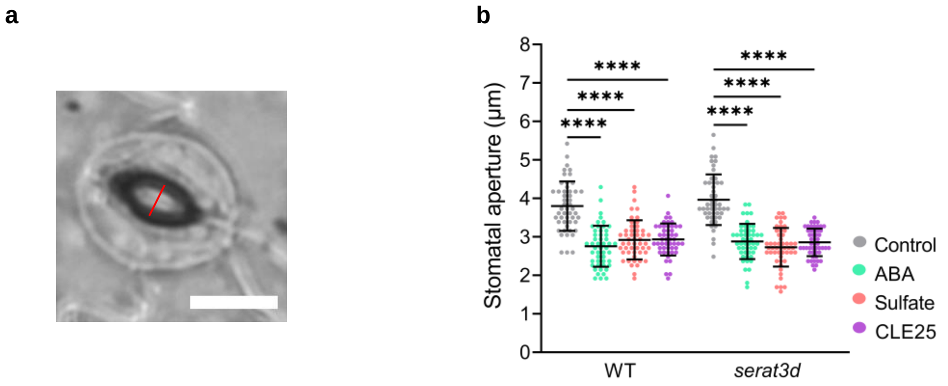

Supplementary Fig. 1 SERAT3s is not required for soil-drying signals (sulfate and CLE25)-induced stomatal closure.

a, Representative image of stomata embedded in an epidermal peel. Red lines indicate the position used for measuring the stomata aperture. The typical aperture value for open stomata using this method is 3–5 µm. Scale bar, 10 µm. **b**, Stomatal apertures of WT and *serat3d* double mutant (*serat3;1serat3;2*) grown for 7 weeks on soil under short day conditions. Isolated stomata in epidermal peels were treated with water (control), 50 µM ABA for 1 h, 15 mM sulfate for 3 h, or 1 µM CLE25 for 3 h (n = 50). The data represent results from two independent experiments and are shown as mean ± SD. Statistical differences were analyzed by one-way ANOVA followed by Tukey's test. ****P < 0.0001.

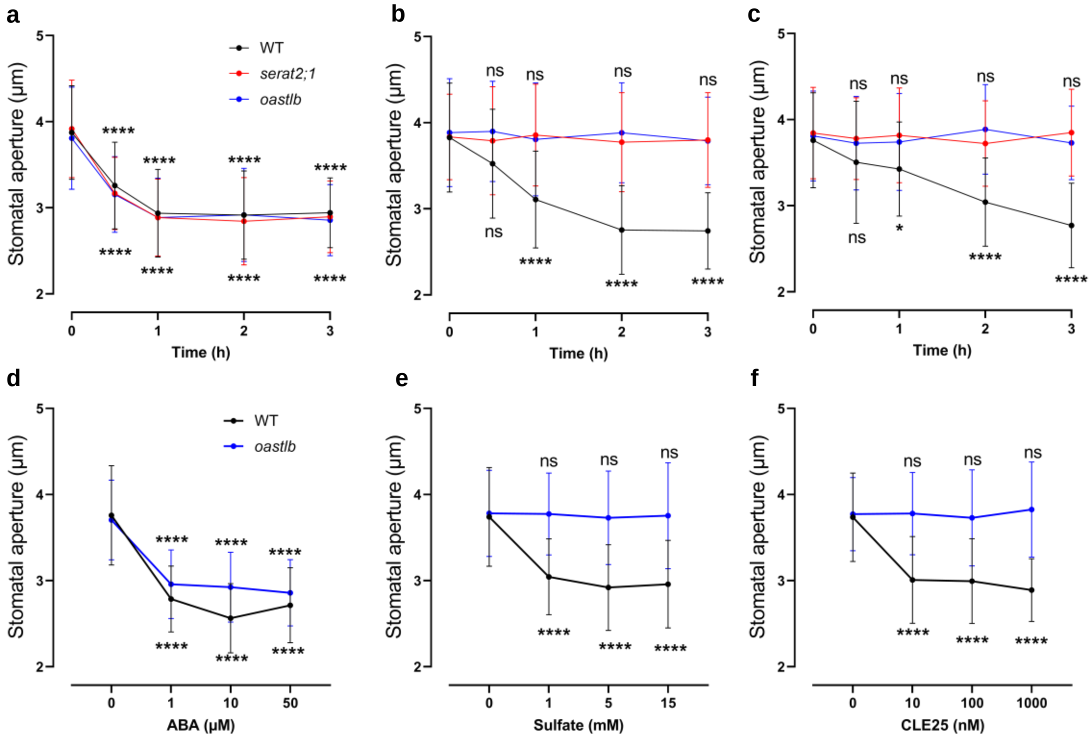

Supplementary Fig. 2 Time course and dose effect of ABA, sulfate, and CLE25 on stomata from wild type (WT) and pCSC mutants.

a-c, Time course analysis (0, 0.5, 1, 2, 3 h) of stomatal apertures of wild type (WT) and mutants lacking the pCSC (*serat2;1* and *oastlb*) in response to ABA (**a**), sulfate (**b**), or the peptide hormone CLE25 (**c**). Isolated stomata in epidermal peels were treated with water (control), 50 µM ABA, 15 mM sulfate or 1 µM CLE25 for indicated time points (n = 50). **d-f**, Stomatal apertures of the WT and the *oastlb* mutant in response to different concentration of ABA (**d**), sulfate (**e**) and CLE25 (**f**). Isolated stomata in epidermal peels were treated with increasing concentrations of ABA (1, 10, 50 µM) for 1 h, sulfate (1, 5, 15 mM) for 3 h, or the peptide hormone CLE25 (10, 100, 1000 nM) for 3 h, respectively (n = 50). Data in **a-f** are shown as mean ± SD. Statistical differences were analyzed by two-way ANOVA followed by Tukey's test, compare to relative control (time point 0). *$P < 0.05$. ****$P < 0.0001$. ns, no significant difference.

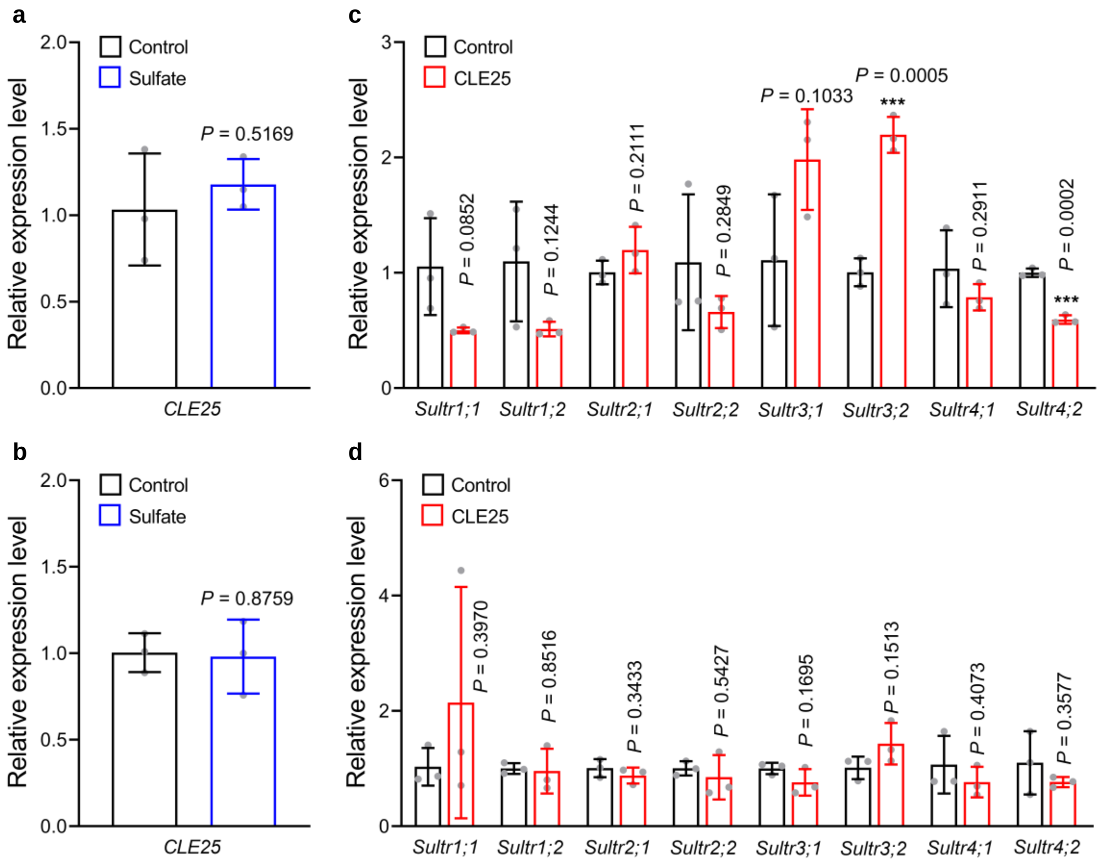

Supplementary Fig. 3 Relative expression levels of *CLE25* and sulfate transporter (*Sultr*) genes. **a**,**b**, Relative expression levels of *CLE25* gene in roots (**a**) and leaves (**b**) of wild type (WT) plants as determined by RT-qPCR. WT plants were grown in ½ Hoagland solution for six weeks and then treated with water (control) or 15 mM Sulfate for 3 h. Relative steady state transcript levels were determined by RT-qPCR. **c**,**d**, Relative expression levels of sulfate transporter (*Sultr*) genes in roots (**c**) and leaves (**d**) of wild type (WT) plants. WT plants were grown in ½ Hoagland solution for six weeks and then treated with water or 1 µM CLE25 for 3 h. The transcripts were measured by RT-qPCR using primers defined in Supplementary Table 1 and *PP2A* as the reference gene. Data are shown as mean ± SD (n = 3). Data were analysed by two-tailed Student's *t*-test. ****P* < 0.001.

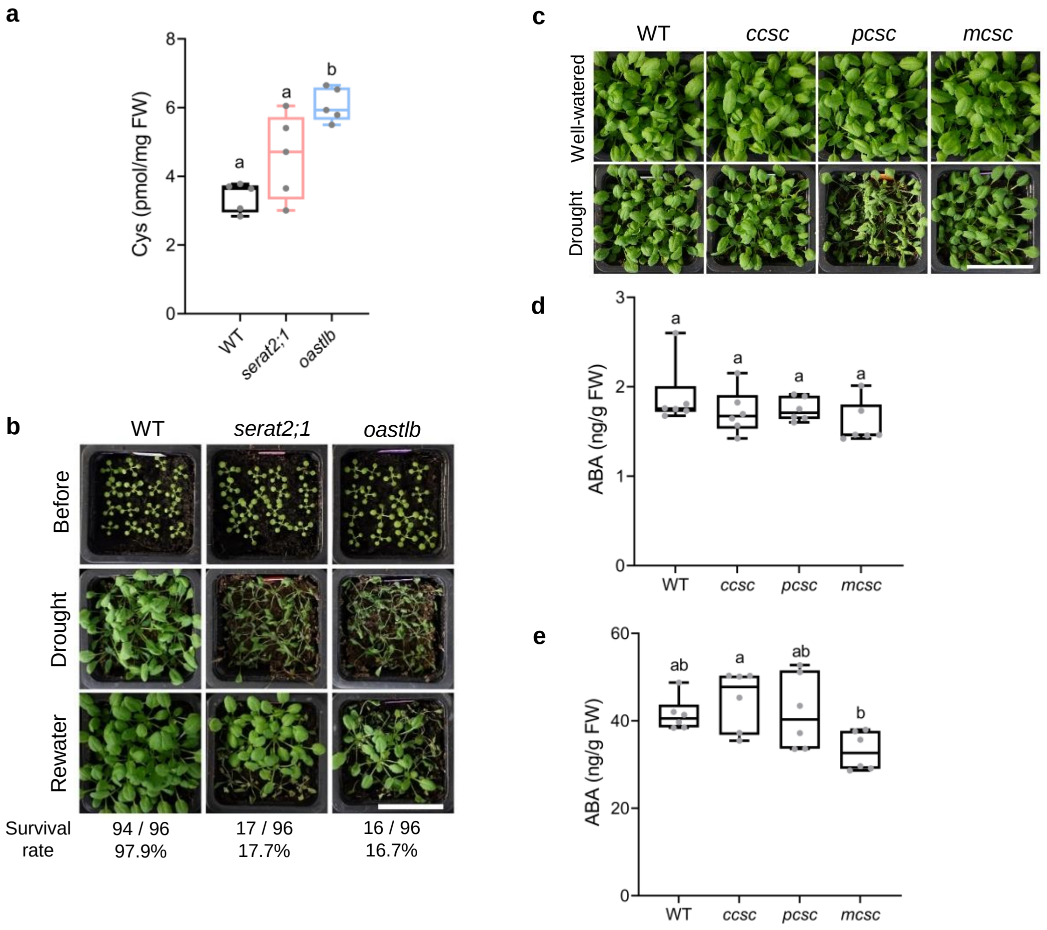

Supplementary Fig. 4 The pCSC is essential for soil-drying signals-induced ABA response and stomatal closure.

a, Cys concentration in leaves of the wild type (WT) and mutants lacking the pCSC (*serat2;1* and *oastlb*). Data are shown as boxplot (n= 5). The box plots display medians (horizontal lines), 25% to 75% ranges (boxes) and min to max (whiskers). **b**, The pCSC mutants *serat2;1* and *oastlb* are sensitive to soil-drying. 18-days-old seedlings of the WT and pCSC mutants were subjected to water withdrawal for 15 days. Representative images were taken before drought (before), at the end of water withdrawal (drought), and 3 days after rewatering (rewater). The survival rate of genotypes is based on subjecting 96 individuals to water limitation. Scale bar, 5 cm. **c**, Exclusively, mutants lacking both pCSC subunits (*pcsc*, *serat2;1oastlb* double mutant) are sensitive to soil-drying. 19-days-old seedlings of the WT and mutants lacking both subunits of the CSC located in the cytosol (*ccsc*, *serat1;1oastla* double mutant), the plastid (*pcsc*) and the mitochondria (*mcsc*, *serat2;2oastlc*) were subjected to water withdrawal for 14 days. Representative images were taken for 14-day well-watered plants (well-watered) and 14-day water withdrawal plants (drought). Scale bar, 5 cm. **d**-**e**, ABA concentrations of the leaves of well-watered (**d**) and drought-stressed (**e**) WT and different *csc* mutants showed in **c** (n = 6). Statistical differences were analyzed by one-way ANOVA followed by Tukey's test. Different letters indicate significant differences among different genotypes ($P < 0.05$).

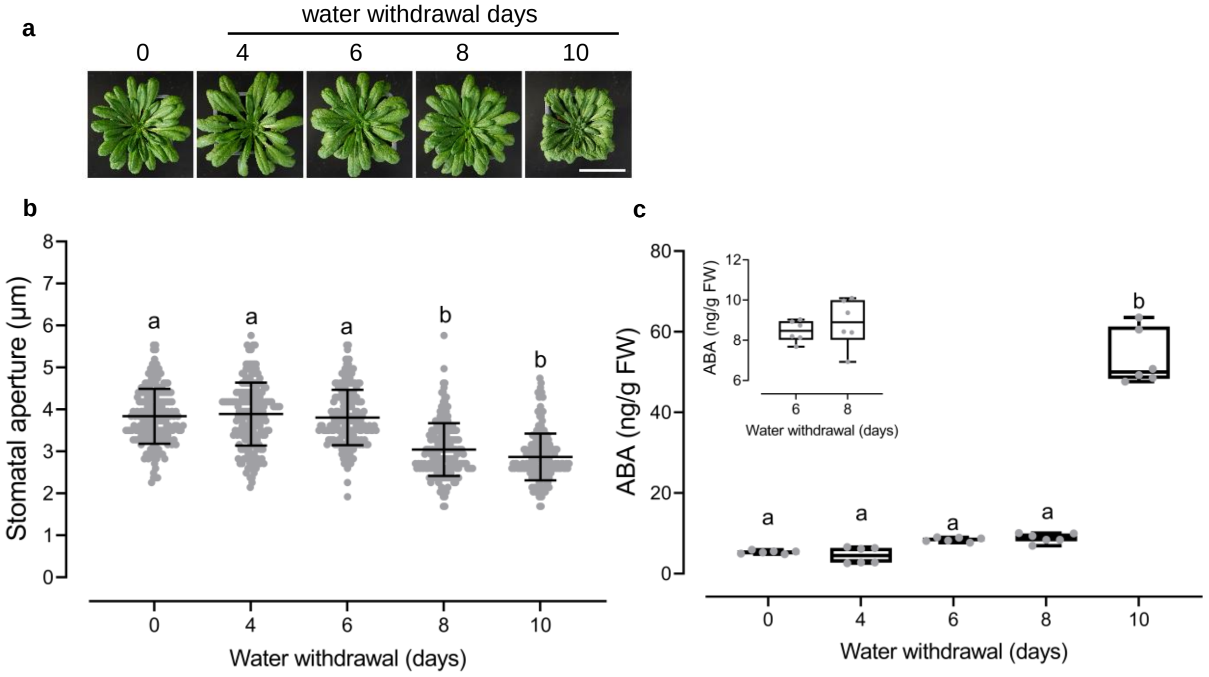

Supplementary Fig. 5 Massive accumulation of ABA in leaves occurs after soil drying-induced stomata closure.

a, Time course of drought stress in wild type (WT, Col-0). 8 week-old WT plants were subjected to water withdrawal for 10 days. Representative images were taken before drought (day 0) and during water withdrawal at days 4, 6, 8, and 10. Scale bar, 5 cm. **b**, Apertures of imprinted stomata from leaves of WT during drought stress. Data are shown as mean ± SD (n = 200). **c**, ABA concentrations of the leaves of WT during drought stress. Data are shown as a box plot (n = 6). The box plots display medians (horizontal lines), 25% to 75% ranges (boxes), and minimum to maximum values (whiskers). Data were analysed by one-way ANOVA followed by Tukey's test. Different letters indicate significant differences among different genotypes ($P < 0.05$).

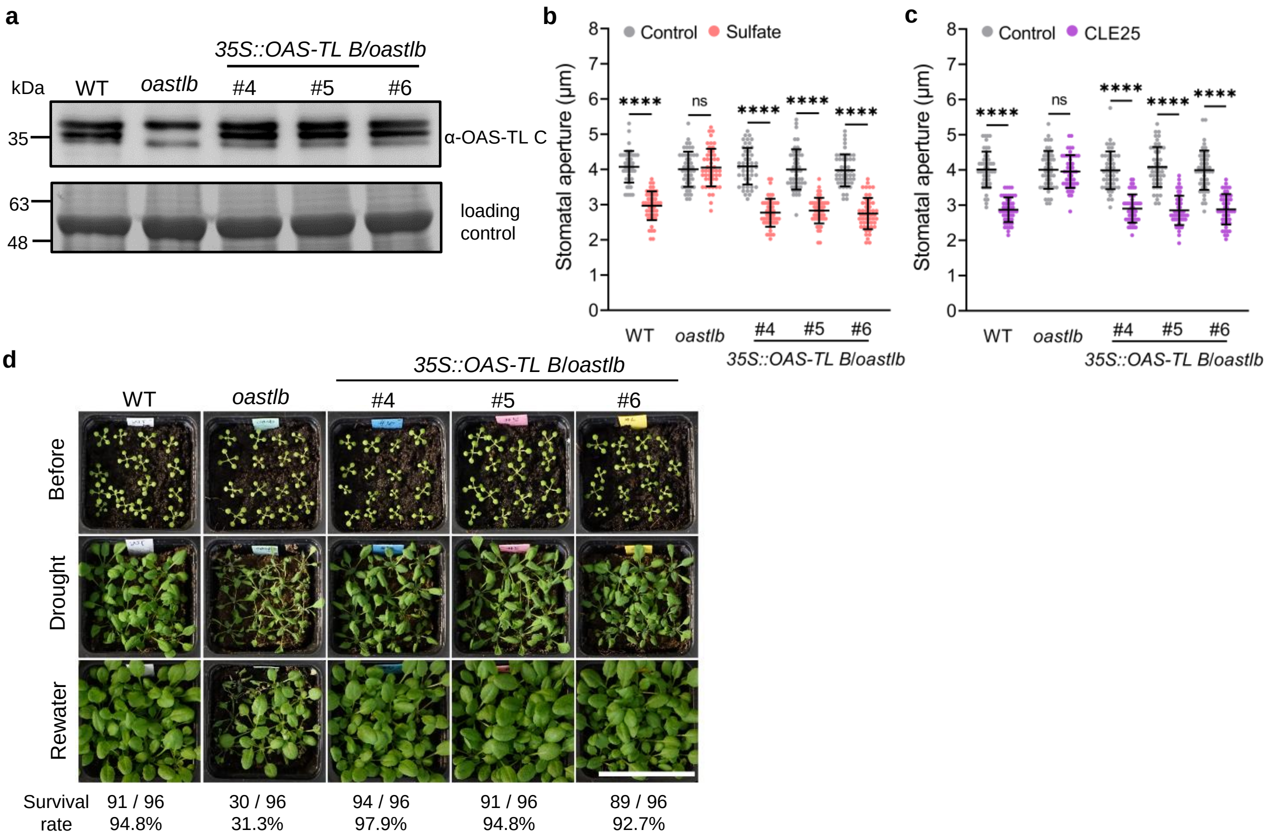

Supplementary Fig. 6 Complementation of *oastlb* with OAS-TL B rescues the loss of soil-drying signals-induced stomata closure and the drought-sensitive phenotype of *oastlb*.
a, Validation of OAS-TL B expression in three independent *p35S::OAS-TLB oastlb* lines by immunoblot analysis. OAS-TL B protein (middle signal) is detected by an antiserum raised against AtOAS-TL C (α-OAS-TLC), which also decorates OAS-TL C (upper signal) and OAS-TL A (lower signal). Comassie Brilliant Blue staining of Rubisco large subunit is used as a loading control. **b**-**c**, Apertures of stomata embedded in epidermal peels of wild type (WT, Col-0), *oastlb* and the three *35S::OAS-TL B* complemented *oastlb* lines treated with sulfate (**b**), or CLE25 (**c**). Data in **b** and **c** are representative results from two independent experiments. Data are shown as mean ± SD (n = 50). Data were analysed by one-way ANOVA followed by Tukey's test. ****P < 0.0001. ns, no significant difference. **d**, Expression of OAS-TLB rescues the soil-drying sensitive phenotype of *oastlb*. 18-days-old seedlings of the WT, *oastlb,* and three *35S::OAS-TL B* complemented *oastlb* lines were subjected to water withdrawal for 16 days. Representative images were taken before drought (before), at the end of water withdrawal (drought), and 3 days after rewatering (rewater). The survival rate of each genotypes is based on the results of subjecting 96 individuals to water limitation. Scale bar, 5 cm.

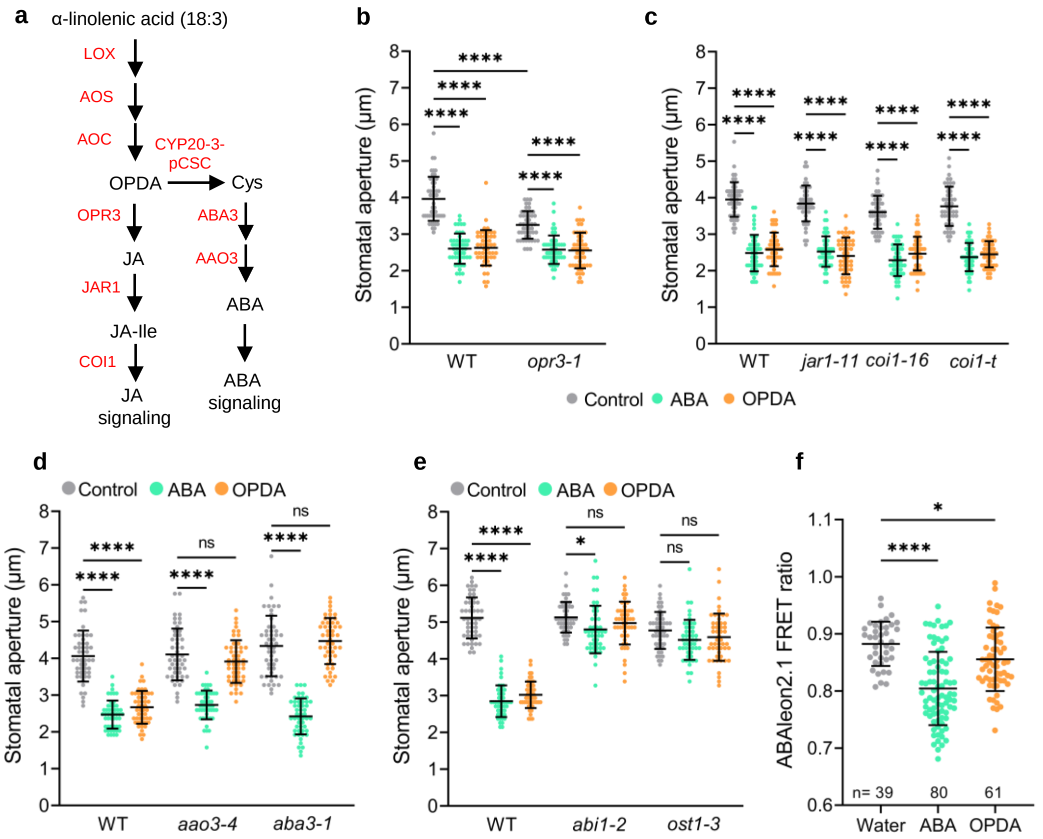

Supplementary Fig. 7 OPDA-induced stomatal closure requires CYP20-3-pCSC based ABA biosynthesis and signaling pathway, rather than JA signaling pathway.
a, Schematic diagram of OPDA-related JA and ABA pathway in Arabidopsis. OPDA is the precursor of JA. OPDA stimulating CYP20-3-SERAT2.1 interaction and plastid CSC formation to produce Cys. **b**, Apertures of stomata embedded in epidermal peels of wild type (WT, Ws) and *opr3-1* mutant treated with water (control, grey), ABA (green), or OPDA (orange). The epidermal peels were pre-treated with an opening buffer (50 mM KCl, 10 mM MES, pH 5.5) for 2 h to ensure that stomata were open at the beginning of the effector treatment (n= 50). **c**, Apertures of stomata embedded in epidermal peels of WT (Col-0) and mutants affected in JA-Ile formation (*jar1-11*) or JA-Ile signalling (*coi1-16*, *coi1-t*) that were treated with water (control), ABA, or OPDA (n = 50). **d**, Apertures of stomata embedded in epidermal peels of WT and two ABA biosynthesis mutants (*aao3-4*, *aba3-1*) treated with water (control), ABA or OPDA (n= 50). **e**, Apertures of stomata embedded in epidermal peels of WT and two ABA signaling mutants (*abi1-2*, *ost1-3*) treated with water (control), ABA or OPDA (n= 50). Data in **b**-**e** are representative results from two independent experiments. **f**, OPDA triggers ABA biosynthesis in guard cells. ABAleon2.1 sensor emission ratio as a proxy for ABA in guard cells after treatment with water, ABA, or OPDA (n = 39-80). Data in **b**-**f** are shown as mean ± SD. Data were analysed by one-way ANOVA followed by Tukey's test (**b**-**e**) or Dunnett's test (**f**). ****$P < 0.0001$. ***$P < 0.001$. *$P < 0.05$. ns, no significant difference.

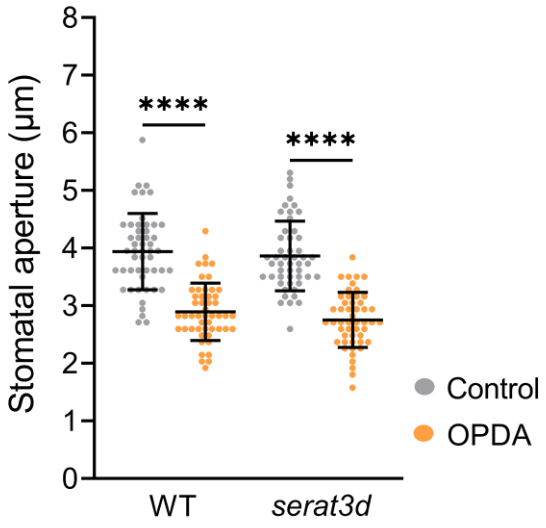

Supplementary Fig. 8 SERAT3s is not required for OPDA-induced stomatal closure.
Stomatal apertures of WT and serat3d double mutant (*serat3;1serat3;2*). Isolated stomata in epidermal peels were treated with water (control), or 10 μM OPDA for 2 h (n = 50). Data are representative results from two independent experiments. Data are shown as mean ± SD. Statistical differences were analyzed by one-way ANOVA followed by Tukey's test. ****P < 0.0001.

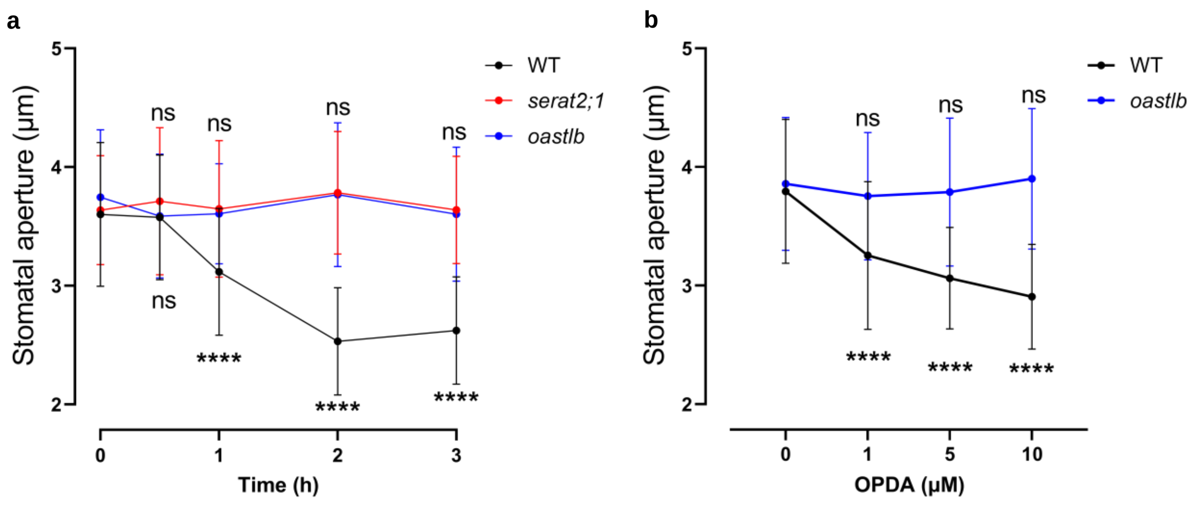

Supplementary Fig. 9 Time course and dose effect of stomatal aperture response to OPDA in wild type (WT) and pCSC mutants.

a, Time course analysis (0, 0.5, 1, 2, 3 h) of stomatal apertures of WT and mutants lacking the pCSC (*serat2;1* and *oastlb*) in response to OPDA. Isolated stomata in epidermal peels were treated with water (control, point 0), 10 µM OPDA (n = 50). **b**, Stomatal apertures of WT and *oastlb* mutant in response to different concentrations of OPDA. Isolated stomata in epidermal peels were treated with water (point 0), or increasing concentrations of OPDA (1, 5, 10 µM) for 2 h (n = 50). Data in **a-b** are shown as mean ± SD. Statistical differences were analyzed by two-way ANOVA followed by Tukey's test, compare to relative control (0 µM OPDA). ****$P < 0.0001$. ns, no significant difference.

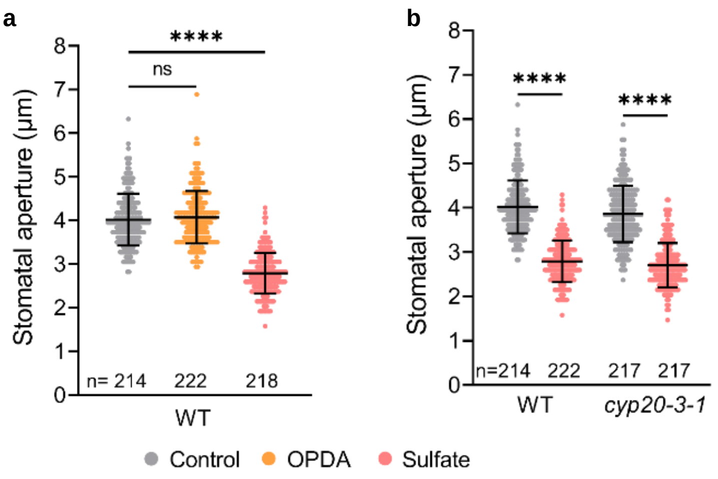

Supplementary Fig. 10 Petiole feeding experiments of OPDA and sulfate treatments.
a, Apertures of imprinted stomata from detached leaves of WT (Col-0) after feeding of water (control), OPDA (orange), or sulfate (red) via the petiole (n= 214-222). **d**, Apertures of imprinted stomata from detached leaves of WT (Col-0) and *cyp20-3-1* mutant after feeding of water (control) or sulfate via the petiole (n= 214-222). Data in **a**-**b** are shown as mean ± SD and were analysed by one-way ANOVA followed by Tukey's test (**a**-**b**). ****$P < 0.0001$. ns, no significant difference.

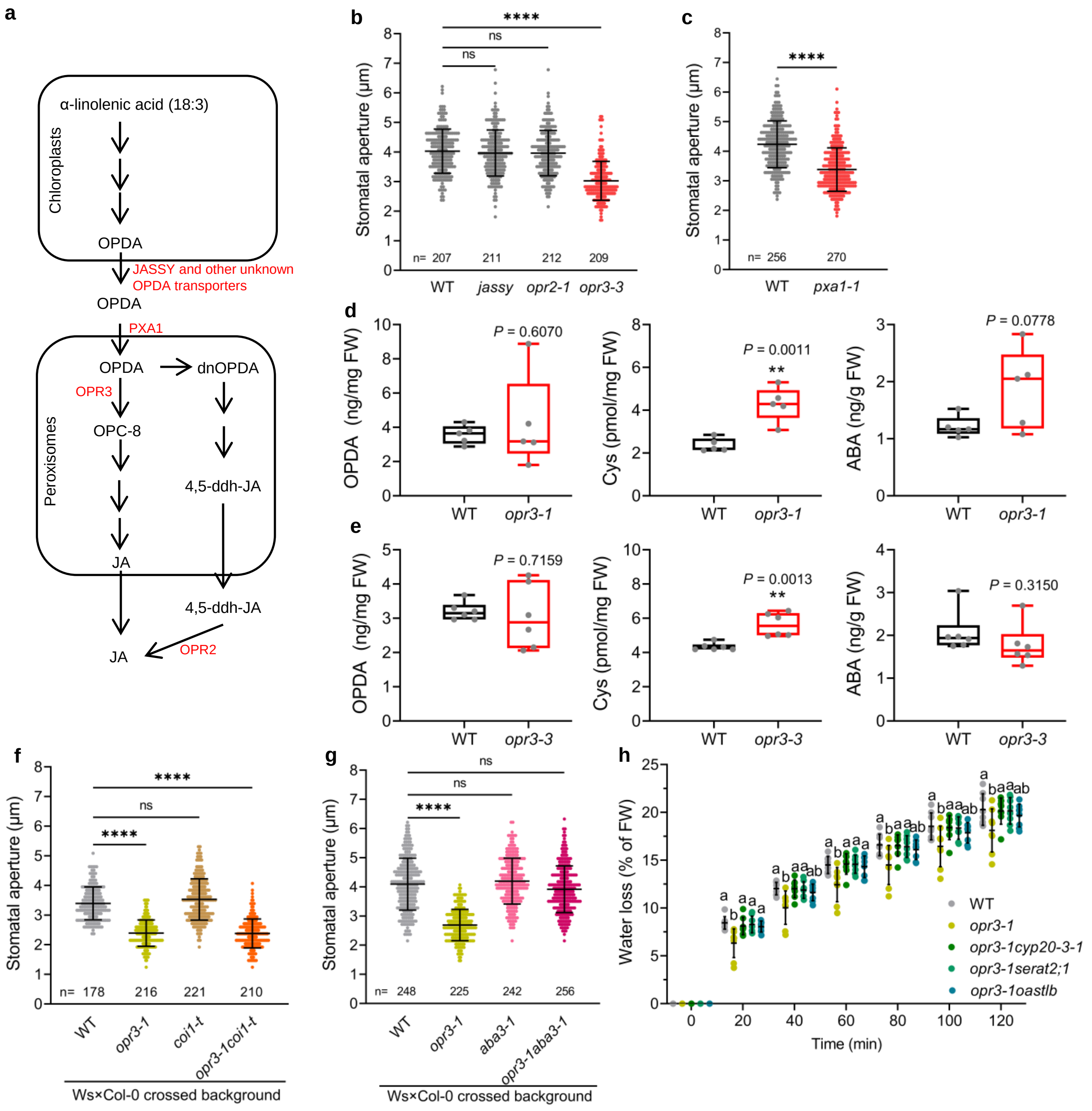

Supplementary Fig. 11 *opr3* mutant displays closed stomata and reduced water loss and it rescued by blocking plastid CSC-based ABA biosynthesis pathway.

a, Schematic diagram of the subcellular localization of the JA biosynthesis pathway in Arabidopsis. OPDA is produced in the chloroplast stroma and then transported over the inner envelope membrane into the intramembrane space of the plastids by an unknown transport system. The transport from the intramembrane system to the cytosol is facilitated by JASSY and other unknown transporter systems. **b**, Apertures of imprinted stomata from leaves of wild type (WT, Col-0), *jassy*, *opr2-1* and *opr3-3* mutants (n = 207-212). **c**, Apertures of imprinted stomata from leaves of WT and *pxa1-1* mutant (n = 256-270). **d**, OPDA, Cys, and ABA levels in leaves of WT (Ws) and *opr3-1* mutant grown on soil under short day conditions (n = 5). **e**, OPDA, Cys, and ABA levels in leaves of WT (Col-0) and *opr3-3* mutant grown on soil under short day conditions (n = 6). **f**, Apertures of imprinted stomata from leaves of WT, *opr3-1*, *coi1-t* or *opr3-1coi1-t* mutant grown on soil under short day conditions (n = 178-221). **g**, Apertures of imprinted stomata from leaves of WT, *opr3-1*, *aba3-1* or *opr3-1aba3-1* mutant grown on soil under short day conditions (n = 242-256). **h**, Water loss of rosette leaves of WT, *opr3-1*, *opr3-1cyp20-3*, *opr3-1serat2;1* or *opr3-1oastlb* mutants. Data from two independent experiments (n=8). Data in **b**-**c**, **f**-**h** are shown as mean ± SD. Data in **d**, **e** are shown as boxplot. The box plots display medians (horizontal lines), 25% to 75% ranges (boxes) and min to max (whiskers). Data in **c**, **d**, **e** were analysed by two-tailed Student's *t*-test. Data in **b**, **f-g** were analysed by one-way ANOVA followed by Tukey's test. Data in **h** were analysed by two-way ANOVA followed by Tukey's test. Different letters indicate significant differences among different genotypes ($P < 0.05$). $^{**}P < 0.01$. $^{****}P < 0.0001$. ns, no significant difference.

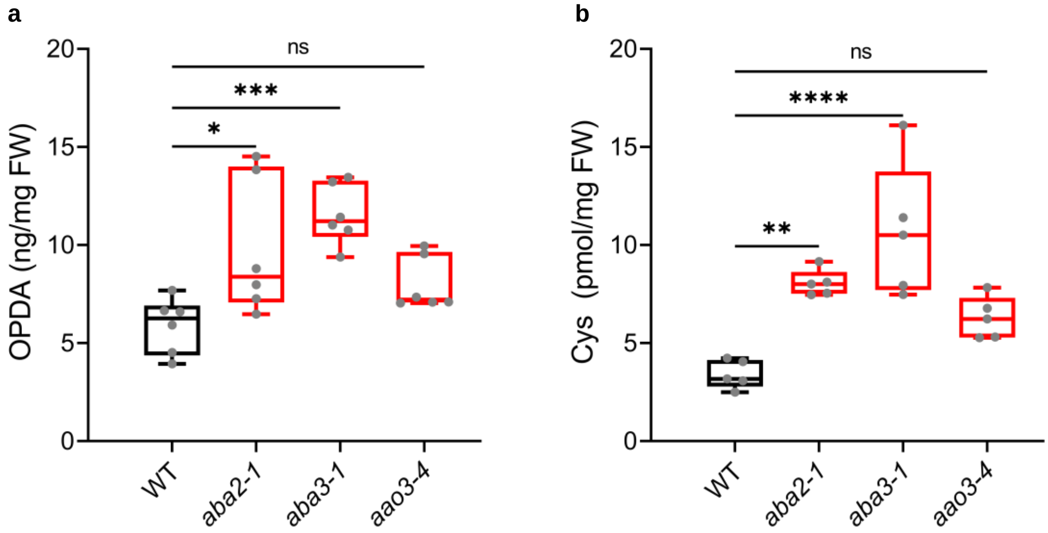

Supplementary Fig. 12 OPDA and Cys concentration in ABA deficiency mutants.
a,**b,** OPDA (**a**) and Cys (**b**) concentrations of WT (Col-0), *aba2-1*, *aba3-1*, and *aao3-4* grown on soil under short-day conditions. Data are shown as boxplot (n= 6 for OPDA, n= 5 for Cys). The box plots display medians (horizontal lines), 25% to 75% ranges (boxes) and min to max (whiskers). Data were analysed by one-way ANOVA followed by Tukey's test. ****$P < 0.0001$. ***$P < 0.001$. **$P < 0.01$. *$P < 0.05$. ns, no significant difference.

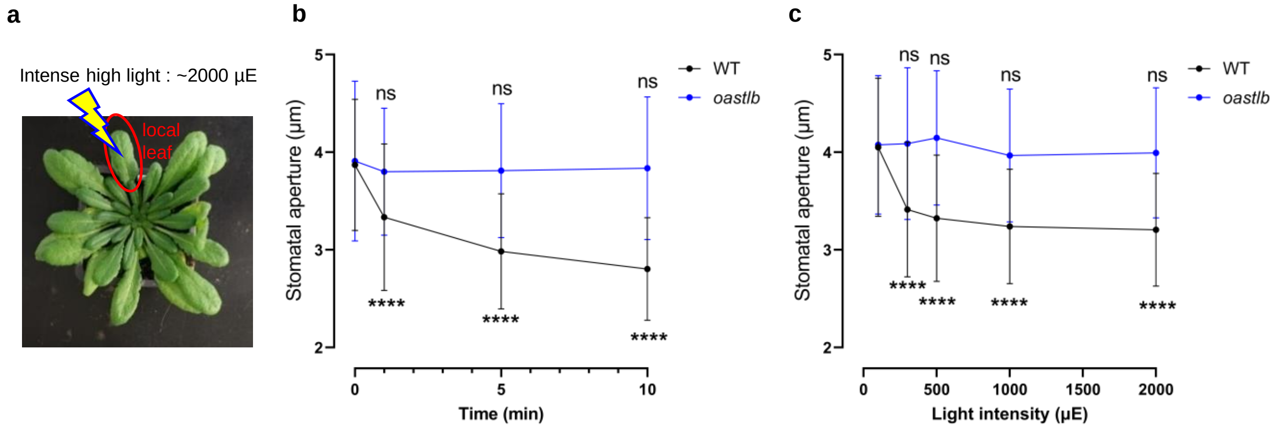

Supplementary Fig. 13 Time course and dose effect of stomatal aperture response to high light in wild type (WT) and pCSC mutants.

a, Definition of intense high light treatment. **b**, Apertures of imprinted stomata from leaves of WT and *oastlb* mutant after high light treatment (2000 µE) for 0, 1, 5, or 10 min (n = 200). Data are shown as mean ± SD. Statistical differences were analyzed by two-way ANOVA followed by Tukey's test. Statistical differences compared to the control (time point 0) are indicated. **b**, Apertures of imprinted stomata from leaves of WT and *oastlb* mutant after different light intensity treatment (100, 300, 500, 1000, 2000 µE) for 10 min (n = 200). Data are shown as mean ± SD. Statistical differences were analyzed by two-way ANOVA followed by Tukey's test, compare to control (100 µE). ****$P < 0.0001$. ns, no significant difference.

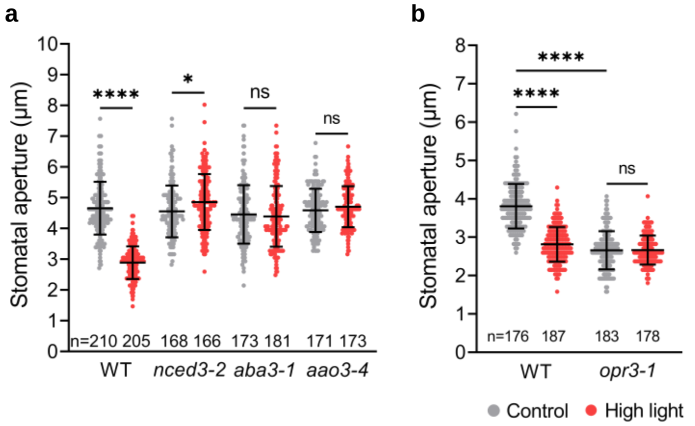

Supplementary Fig. 14 High light-induced stomatal closure requires the synthesis and perception of ABA and JA.

a, Apertures of imprinted stomata from leaves of wild type (WT, Col-0), and three ABA biosynthesis mutants (*nced3-2*, *aba3-1* and *aao3-4*) that were treated with growth light (100 µE, grey) or high light (2000 µE, red) for 10 min (n = 166-205). **b**, Apertures of imprinted stomata from leaves of WT (Ws), and JA biosynthesis mutant (*opr3-1*) that were treated with growth light (100 µE, grey) or high light (2000 µE, red) for 10 min(n = 176-187). **c**, Apertures of imprinted stomata from leaves of WT (Col-0), and three JA biosynthesis and signalling mutants (*aos*, *jar1-11* and *coi1-16*) that were treated with growth light (100 µE, grey) or high light (2000 µE, red) for 10 min (n = 170-207). Data in **a-c** are shown as mean ± SD. Data were analysed by one-way ANOVA followed by Tukey's test. ****P < 0.0001. *P < 0.05. ns, no significant difference.

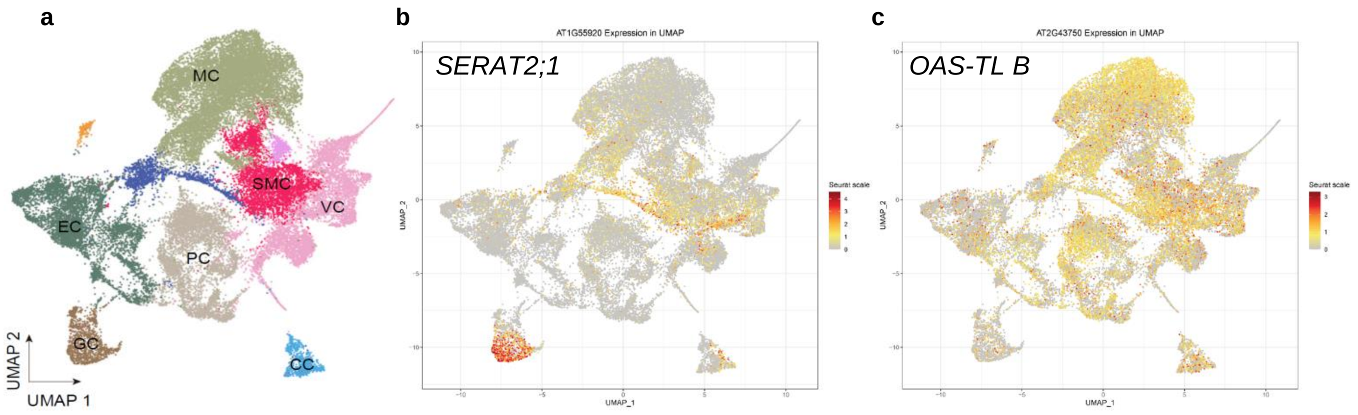

Supplementary Fig. 15 Identification of genes expression from single-cell analysis database.
a, Standard visualization of cell type using the UMAP method according to Zhang *et al.* (2021). GC, guard cell. EC, epidermal cell. MC, mesophyll cell. SMC, shoot meristematic cell. PC, proliferating cell. VC, vascular cell. CC, companion cell. **b-c**, Single-cell gene expression of *SERAT2;1* (AT1G55920, **b**), and *OAS-TL B* (AT2G43750, **c**) according to published database. The expression of genes is visualized with the Arabidopsis Shoot Apex Cell Atlas database (http://wanglab.sippe.ac.cn/shootatlas/).

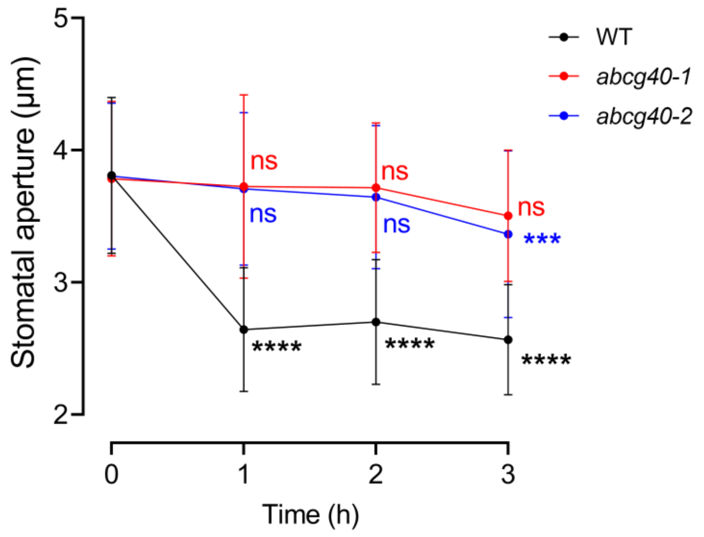

Supplementary Fig. 16 Time course of stomatal aperture response to ABA in wild type (WT) and *abcg40* mutants.

Time course (0, 1, 2, 3 h) of stomatal apertures of WT and two guard cell-localized ABA importer *abcg40* mutants in response to ABA. Isolated stomata in epidermal peels were treated with water (control, point 0), and 50 µM ABA for the indicated time (n = 50). Data were analysed by two-way ANOVA followed by Tukey's test. ****$P < 0.0001$. ns, no significant difference.

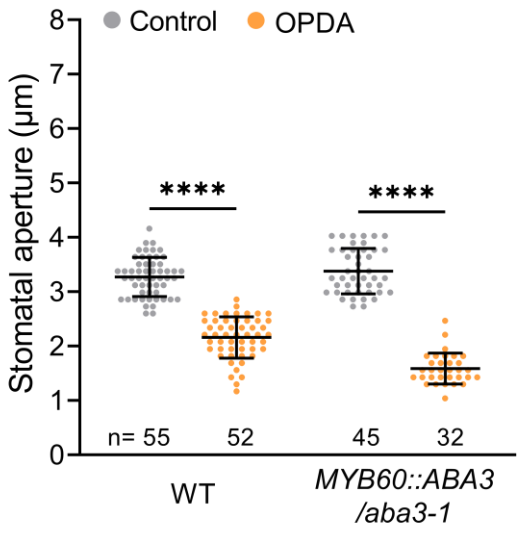

Supplementary Fig. 17 Guard-cell autonomous ABA synthesis in the *MYB60::ABA3 aba3-1* complemented line is sufficient for OPDA-induced stomatal closure.
Apertures of stomata embedded in epidermal peels of WT (Col-0) and *MYB60::ABA3 aba3-1* complemented line treated with water (control), or OPDA (n= 32-55). Data were analysed by one-way ANOVA followed by Tukey's test. ****P < 0.0001.

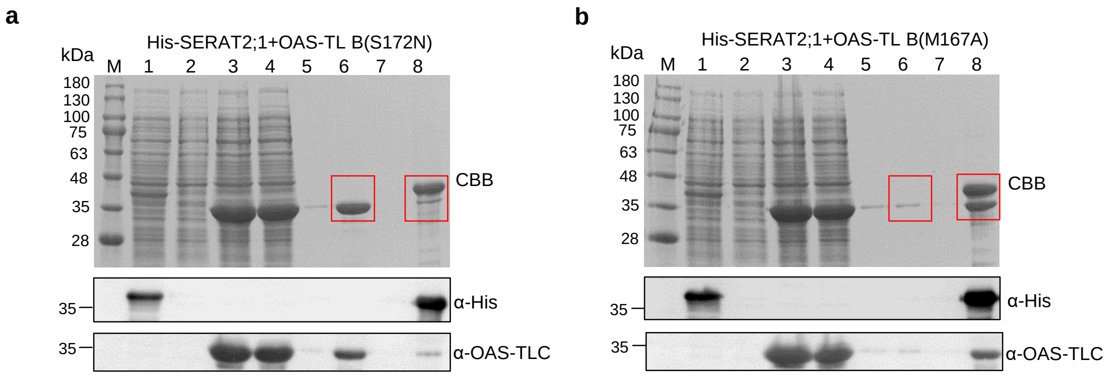

Supplementary Fig. 18 In vitro analysis of the OAS dissociation effect on pCSC.
a,**b**, Fractions of selected protein purification steps (lanes 1-8) from His-SERAT2;1 and OAS-TL B(S172N) (**a**), or His-SERAT2;1 and OAS-TL B(M167A) (**b**) were analyzed by SDS-PAGE and visualized by Coomassie Brilliant Blue (CBB) staining and immunoblot. M: marker; lane 1: crude extract of His-SERAT2;1 expressing bacteria; lane 2: flow through of IMAC column; lane 3: crude extract of bacteria expressing OAS-TL B(S172N) (**a**), or OAS-TL B(M167A) protein (**b**); lane 4: flow through of His-SERAT2;1 loaded column after application of OAS-TL B(S172N) (**a**), or OAS-TL B(M167A) protein (**b**); lane 5: washing buffer; lane 6: OAS elution; lane 7: washing buffer; lane 8: final elution of His-SERAT2;1 and its interaction partners after OAS treatment. Parts of the images labelled with red boxes are shown in Fig. 4a. OAS-TL B(S172N) and OAS-TL B (M167A) bind well to SERAT2;1 immobilized via the His on the Hi-Trap column. Only OAS-TL B(S172N) can be selectively eluted by application of OAS, demonstrating that the OAS-TL B(S172N) containing pCSC is unstable. In contrast, the OAS-TL B(M167A) containing pCSC is resistant to dissociation by OAS, resulting in a constitutive activation of SERAT2;1.

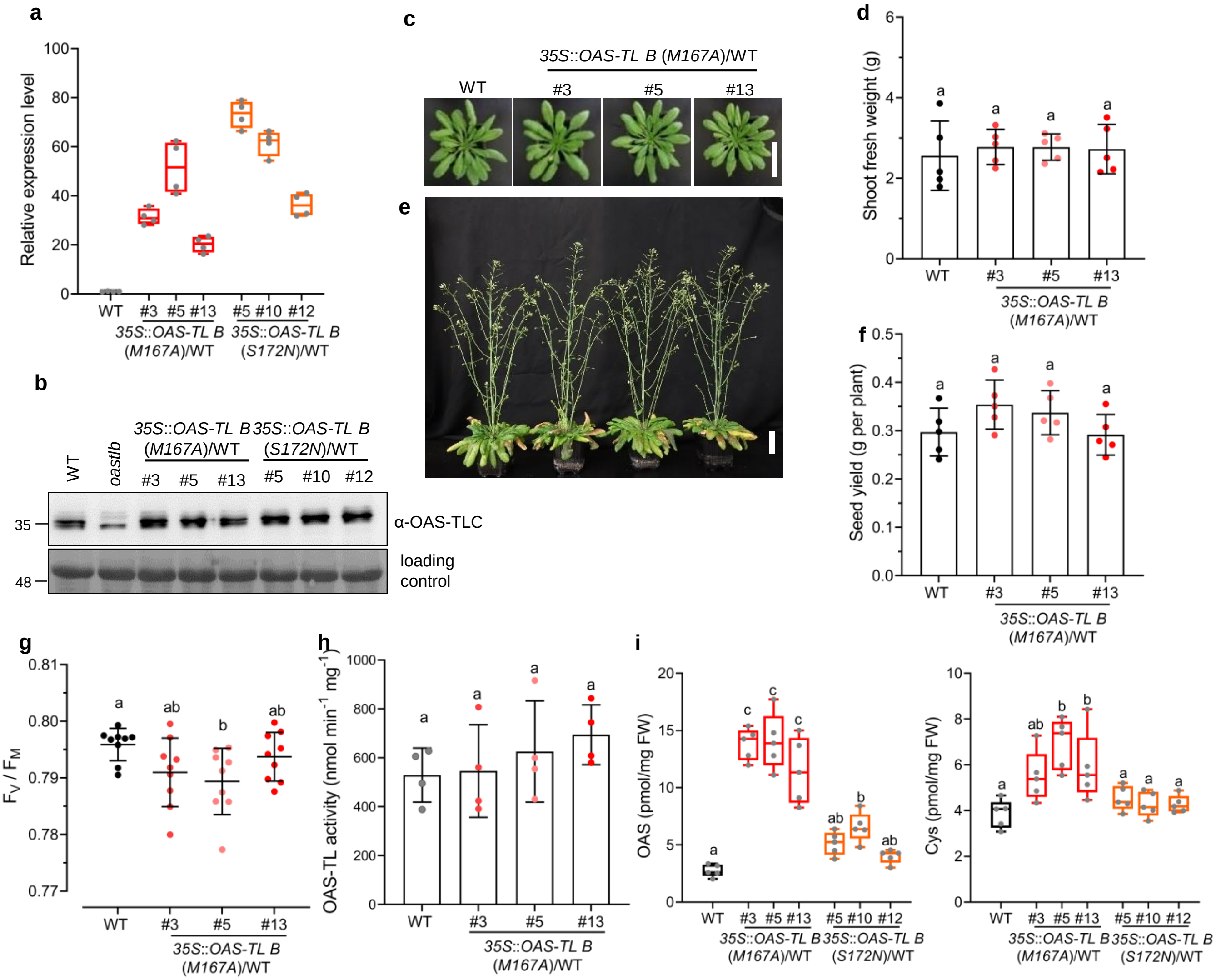

Supplementary Fig. 19 Characterization of constitutively activated pCSC transgenic plants.
a, Relative steady-state transcript levels of *OAS-TL B* gene in wild type (WT), *35S*::*OAS-TL B*(*M167A*) and *35S*::*OAS-TL B*(*S172N*) plants. The transcripts were measured by RT-qPCR, using primers defined in Supplemental Table 1 and *PP2A* as a reference. **b**, Immunological detection of OAS-TL B protein in WT, *oastlb*, *35S*::*OAS-TL B*(*M167A*) and *35S*::*OAS-TL B*(*S172N*) transgenic plants using immunoblot analysis. OAS-TL B protein (middle band) is detected by an antibody raised against AtOAS-TL C (α-OAS-TLC). Amido black staining of the Rubisco large subunit served as a loading control. **c**, **d**, Growth phenotype (**c**) and shoot fresh weight (**d**) of WT, and *35S*::*OAS-TL B*(*M167A*) transgenic plants grown on soil for 8 weeks under short-day conditions. Scale bar, 5 cm. **e**, Growth phenotype of 12-week-old WT, and *35S*::*OAS-TL B*(*M167A*) transgenic plants grown on soil. Plants were grown in short day condition for 9 weeks and then transfer to long day condition for 3 weeks to induce flowering. Scale bar, 5 cm. **f**, Seeds yield of WT, and *35S*::*OAS-TL B*(*M167A*) transgenic plants. **g**, Chlorophyll fluorescence (F_v/F_m ratio) of 7-week-old soil-grown WT, and *35S*::*OAS-TL B*(*M167A*) transgenic plants. **h**, Total OAS-TL enzyme activity in whole soluble protein extracts of the leaves of 7-week-old WT, and *35S*::*OAS-TL B*(*M167A*) plants grown on soil under short-day conditions. **i**, OAS and Cys concentrations in leaves of WT, *35S*::*OAS-TL B*(*M167A*) and *35S*::*OAS-TL B*(*S172N*) plants grown on soil under short-day conditions for 7 weeks. Data in **a**, **i** are shown as boxplot (n= 4 in **a**, n= 5 in **i**). The box plots display medians (horizontal lines), 25% to 75% ranges (boxes) and min to max (whiskers). Data in **d**, **f-h** are shown as mean ± SD (n= 5 in **d**, **f**, n= 9 in **g**, n= 4 in **h**). Data were analysed by one-way ANOVA followed by Tukey's test. Different letters indicate significant differences among different genotypes (P < 0.05).

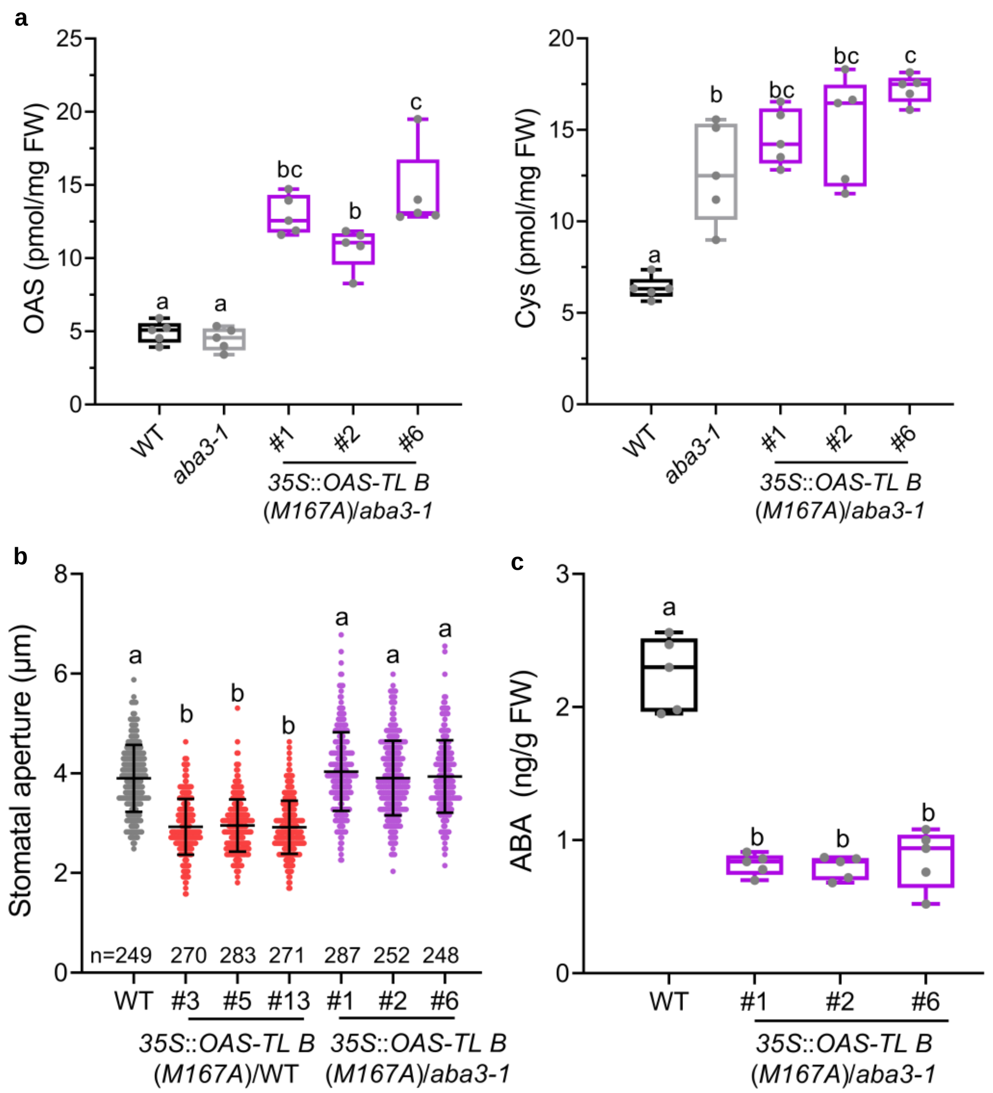

Supplementary Fig. 20 Constitutively activated pCSC induced stomatal closure is dependent on ABA biosynthesis.

a, Steady-state levels of OAS and Cys in leaves of wild type (WT), *aba3-1*, and three independent *aba3-1* lines expressing *OAS-TL B(M167A)* that were grown on soil for 7 weeks under short-day conditions. **b**, Apertures of imprinted stomata from leaves of 7-week-old soil grown WT and transgenic plants expressing OAS-TL B(M167A) in the WT or *aba3-1* (n = 248-287). A representative result of two independent experiments is shown as mean ± SD. **c**, Steady-state level of ABA in the leaves of WT and three independent aba3-1 lines expressing OAS-TL B(M167A). Plants were grown on soil for 7 weeks under short-day conditions. Data in **a**, **c** are shown as boxplot (n = 5). The box plots display medians (horizontal lines), 25% to 75% ranges (boxes) and min to max (whiskers). Data were analysed by one-way ANOVA followed by Tukey's test (**a**-**c**). Different letters indicate significant differences among different genotypes ($P < 0.05$).

Supplementary Table 1 Primers used for plasmid construction and qRT-PCR analysis.

Primer Name	Forward oligonucleotide (5'-3')	Reverse oligonucleotide (5'-3')	Purpose
OAS-TLB-CDS	ATGGCGGCGACATCTTCCTC	TCAAAGCTCGGGCTGCATTTG	To PCR amplify CDS regeion of OAS-TL B
OAS-TLBΔN60	GCTGTATCTATCAAGCCAGAAGCTG	TCAAAGCTCGGGCTGCATTTGCTC	To PCR amplify CDS regeion of OAS-TL B without 60 aa in N-terminus
OAS-TLB (M167A)	CTTATCTTGACGGCGCCTGCGTC	GACGCAGGCGCCGTCAAGATAAG	To point mutation
OAS-TLB (S172N)	CCTGCGTCCATGAATTTGGAAAG	CTTTCCAAATTCATGGACGCAGG	To point mutation
OAS-TLB (S172N)-pMDC32	GGGGACAAGTTTGTACAAAAAAGCAGGCTATGGCGGCGACATCTTCCT	GGGGACCACTTTGTACAAGAAAGCTGGGTTCAAAGCTCGGGCTGCATTTG	To clone OAS-TL B(S172N) into pMDC32
OAS-TLB (M167A)-pMDC32	AAGGGGCGCGCCATGGCGGCGACATCTTCCTC	TGTTTTAATTAATCAAAGCTCGGGCTGCATTTG	To clone OAS-TL B(M167A) into pMDC32
SERAT2;1-pET30a	AAGGCCATGGCACAAATCGAAGATGACGATGA	ATTCGGATCCTTAAATCACATAATCAGACCACTCG	To clone SERAT2;1 into pET30a
OAS-TLBΔN60-pET30a/3d	AAGGCCATGGCTGTATCTATCAAGCCAGAAGCTGG	TGTTGGATCCTCAAAGCTCGGGCTGCATTTG	To clone OAS-TLBΔN60 into pET30a or pET3d
PP2A-qPCR	CTTCTCGCTCCAGTAATGGGATCC	GCTTGGTCGACTATCGGAATGAGAG	RT-qPCR
OAS-TL B-qPCR	GTGTCGAACCCACGGAAAGT	ACCAACAAGCCTTCCTGGAG	RT-qPCR
CLE25-qPCR	GGTAAGGATGTGAATCTGTTTCATGT	TCTGCTTTCCTGTTGTGGATAGG	RT-qPCR
Sultr1;1-qPCR	CATGCCGGCAATCAGATTTGTG	TTCTTGCTTCTCGAGGCTCTTG	RT-qPCR
Sultr1;2-qPCR	TCGTGTTGACTCCGCCATTTAC	AGGTAGGCTTGCTGCTTTTACC	RT-qPCR
Sultr2;1-qPCR	ATTCGTCCAGGGATAGAAACGC	TGGCATTGGCAAAGCACAAC	RT-qPCR
Sultr2;2-qPCR	ATGACATCCGGTTAGTCATCGC	CTCGTCCAATTTTGCTCGCTTC	RT-qPCR
Sultr3;1-qPCR	GAGCGTTAAAGTTGGTATTGTC	CTATACGTTGTTCCAAGGCTC	RT-qPCR
Sultr3;2-qPCR	TCACTCGCTCTTCTCTTCTCAT	TGTTGAGTTCCTCAAGCATGCT	RT-qPCR
Sultr4;1-qPCR	AATGGTGGAGCTAGTTGGCAAG	TGTTGTTGCTCCCACCATATCG	RT-qPCR
Sultr4;1-qPCR	TTGTTTGGTCTTGGCGTTGC	CAGTCTTGGCTTCACTTTCGC	RT-qPCR
